# 基于单细胞转录组学的新辅助免疫联合化疗治疗后肺鳞癌患者肿瘤免疫微环境特征的初步分析

**DOI:** 10.3779/j.issn.1009-3419.2026.102.05

**Published:** 2026-03-20

**Authors:** Guannan WANG, Yanan WANG, Jinghao LIU, Minghui LIU, Xuanguang LI, Zihe ZHANG, Hongbing ZHANG, Yu HUA, Yongwen LI, Hongyu LIU, Jun CHEN

**Affiliations:** ^1^300052 天津，天津医科大学总医院胸部肿瘤中心，肺部肿瘤外科; ^1^Department of Lung Cancer Surgery, Thoracic Tumor Center, Tianjin Medical University General Hospital; ^2^天津医科大学总医院，天津市肺癌研究所，天津市肺癌转移与肿瘤微环境重点实验室; ^2^Tianjin Key Laboratory of Lung Cancer Metastasis and Tumor Microenvironment, Tianjin Lung Cancer Institute, Tianjin Medical University General Hospital, Tianjin 300052, China

**Keywords:** 肺肿瘤, 单细胞测序, 新辅助治疗, 肿瘤微环境, Lung neoplasms, Single-cell sequencing, Neoadjuvant therapy, Tumor microenvironment

## Abstract

**背景与目的** 新辅助免疫联合化疗是非小细胞肺癌（non-small cell lung cancer, NSCLC）的重要治疗策略，但患者治疗反应异质性显著，目前疗效评估多依赖影像学检查，对治疗后肿瘤微环境（tumor microenvironment, TME）变化及其与疗效的关联仍缺乏足够的研究。本研究应用单细胞RNA测序（single-cell RNA sequencing, scRNA-seq）技术解析新辅助免疫联合化疗后肺鳞癌TME重塑特征，探讨其与疗效差异的潜在关系。**方法** 对2例接受新辅助免疫联合化疗后手术切除的肺鳞癌患者肿瘤组织进行scRNA-seq，整合分析TME的细胞组成、功能状态及细胞间通讯特征。**结果** 术前影像学评估病例1呈现部分缓解（partial response, PR）、病例2为疾病稳定（stable disease, SD）状态；术后病理评估分别为主要病理缓解（major pathological response, MPR）和部分病理缓解（partial pathological response, PPR）。scRNA-seq分析共鉴定出9种主要细胞群。2例患者TME存在明显异质性：病例1呈免疫细胞富集和炎症激活特征；病例2以髓系及内皮信号重塑为主，伴更明显的免疫调节和血管生成特征。细胞通讯分析显示，病例1以炎症趋化相关互作为主，病例2则表现为CXC趋化因子（C-X-C motif chemokine ligand, CXCL）、分泌型磷蛋白1（secreted phosphoprotein 1, SPP1）、血管内皮生长因子（vascular endothelial growth factor, VEGF）、巨噬细胞移动抑制因子（macrophage migration inhibitory factor, MIF）和肿瘤坏死因子（tumor necrosis factor, TNF）等信号增强，并突出免疫抑制相关互作轴；同时，病例2中巨噬细胞相对富集，T细胞耗竭特征更明显。**结论** 新辅助免疫联合化疗后肺鳞癌患者TME在细胞组成、功能状态及细胞通讯层面存在显著异质性，scRNA-seq揭示的TME重塑模式可为理解疗效评估差异、探索潜在生物标志物提供线索。

## Abstract (en)

**Background and objective** Neoadjuvant immunochemotherapy has emerged as an important treatment strategy for non-small cell lung cancer (NSCLC); however, substantial heterogeneity in therapeutic response persists among patients. Currently, treatment efficacy is primarily evaluated by imaging modalities, and there remains a lack of comprehensive studies investigating post-treatment alterations in the tumor microenvironment (TME) and their association with clinical response. This study applied single-cell RNA sequencing (scRNA-seq) to characterize TME remodeling in lung squamous cell carcinoma after neoadjuvant immunochemotherapy and to explore its potential association with differences in therapeutic response. **Methods** Tumor specimens from two patients with lung squamous cell carcinoma who underwent surgical resection following neoadjuvant immunochemotherapy were subjected to scRNA-seq. An integrated analysis was performed to characterize the cellular composition, functional states, and intercellular communication patterns within the TME. **Results** Preoperative imaging evaluation indicated partial response (PR) in patient 1 and stable disease (SD) in patient 2, whereas postoperative pathological evaluation showed major pathological response (MPR) and partial pathological response (PPR), respectively. ScRNA-seq identified nine major cell populations. The TME exhibited marked heterogeneity between the two patients: patient 1 was characterized by immune cell enrichment and inflammatory activation, whereas patient 2 showed remodeling dominated by myeloid and endothelial signals, together with more prominent immune regulatory and angiogenic features. Cell-cell communication analysis revealed that patient 1 was mainly characterized by inflammatory chemotactic interactions, whereas patient 2 showed enhanced C-X-C motif chemokine ligand (CXCL), secreted phosphoprotein 1 (SPP1), vascular endothelial growth factor (VEGF), macrophage migration inhibitory factor (MIF) and tumor necrosis factor (TNF) signaling, along with prominent immunosuppressive interaction axes. In addition, patient 2 showed a relative enrichment of macrophages and a more pronounced exhausted T-cell phenotype. **Conclusion** After neoadjuvant immunochemotherapy, the TME in patients with lung squamous cell carcinoma exhibits marked heterogeneity in terms of cellular composition, functional states, and intercellular communication. The TME remodeling patterns revealed by scRNA-seq may provide insights into discrepancies in treatment response evaluation and help identify potential biomarkers.

肺癌是全球范围内发病率和死亡率均居前列的恶性肿瘤之一，是肿瘤相关死亡的主要负担^[1]^。非小细胞肺癌（non-small cell lung cancer, NSCLC）占全部肺癌病例的80%-85%，其中肺鳞癌作为重要的组织学亚型，在吸烟人群中发生率较高，且常因早期症状不典型而在确诊时已处于局部进展期^[2,3]^。对于这类患者，单纯手术治疗往往难以获得理想的长期预后，围手术期综合治疗逐渐成为临床管理的重要组成部分。

免疫治疗，尤其是免疫检查点抑制剂（immune checkpoint inhibitors, ICIs）给肺癌治疗带来了重大的突破，其核心机制是通过激活患者自身的免疫系统，使其能够识别并攻击肿瘤细胞，具有疗效持久、副作用相对较小等优势^[4]^。随着ICIs在晚期NSCLC中显示出明确的生存获益，将免疫治疗前移至围手术期阶段，尤其是与化疗联合应用，作为可切除II-III期NSCLC的新辅助治疗，以提升病理缓解、提高手术可切除性等优势，成为核心治疗手段^[5]^。目前，美国国立综合癌症网络（National Comprehensive Cancer Network, NCCN）、中国临床肿瘤学会（Chinese Society of Clinical Oncology, CSCO）等指南已经将免疫治疗联合化疗列为II-III期NSCLC的新辅助推荐治疗方案^[6]^。临床研究^[7]^证实，新辅助免疫联合化疗在提高影像学缓解率和病理缓解率方面具有优势，并在可切除NSCLC患者中展现出良好的安全性和可行性。尽管总体疗效得到改善，患者对新辅助免疫治疗的反应仍存在显著个体差异^[8]^。现有研究^[9]^表明，新辅助治疗的效果不仅取决于肿瘤细胞对药物或免疫干预的内在敏感性，还受到肿瘤微环境（tumor microenvironment, TME）中免疫细胞组成、功能状态及细胞间相互作用的深刻影响。然而，大多数临床研究仍以影像学检测作为疗效评判方法，对于治疗后TME在不同疗效状态下的细胞学特征，尤其是免疫生态系统的重塑模式，仍缺乏系统性认识^[10]^。

单细胞RNA测序（single-cell RNA sequencing, scRNA-seq）技术能够在单细胞分辨率下刻画肿瘤及其TME的细胞组成与功能异质性，为解析治疗相关的肿瘤-免疫相互作用提供了有力工具^[11]^。新辅助免疫治疗发生在肿瘤尚未切除、肿瘤抗原库和TME仍相对完整的阶段，这一独特时间窗口使其成为研究治疗诱导的TME重塑过程的理想模型^[12]^。

在此背景下，本研究基于2例接受新辅助免疫治疗后行手术切除的肺鳞癌患者样本，采用scRNA-seq对治疗后肿瘤组织及TME进行分析。通过分析不同样本中上皮细胞、髓系细胞及T细胞的组成特征及潜在的细胞间通讯模式，本研究旨在描绘新辅助免疫治疗后肺鳞癌TME的个体差异特征，系统分析肿瘤细胞和TME的重塑特征，为理解新辅助治疗疗效异质性提供细胞层面的线索。

## 1 资料与方法

### 1.1 患者组织样本采集

本研究共纳入2例经支气管镜活检后病理诊断为肺鳞癌的患者，患者术前均接受了3个周期的新辅助治疗，用药方案为程序性细胞死亡受体1（programmed cell death protein-1, PD-1）抑制剂联合铂类化疗：病例1为卡铂+白蛋白结合型紫杉醇+替雷利珠单抗，病例2为信迪利单抗+顺铂+紫杉醇。2例患者在术前新辅助治疗结束后均接受计算机断层扫描（computed tomography, CT）影像学评估，疗效评估按照实体瘤疗效评价标准1.1（Response Evaluation Criteria in Solid Tumors 1.1, RECIST 1.1）进行。其中病例1符合部分缓解（partial response, PR）标准，病例2符合疾病稳定（stable disease, SD）标准。随后，患者于天津医科大学总医院肺部肿瘤外科接受肿瘤切除手术。手术切除后立即收集新鲜肺肿瘤组织进行组织处理和scRNA-seq。术后肺鳞癌肿瘤组织用特定储存液保存后送上海宏序生物科技公司进行scRNA-seq。术前患者均签署知情同意书。

术后肿瘤切除标本均按照常规临床病理流程进行固定、取材、石蜡包埋及切片处理。连续切片经苏木精-伊红（hematoxylin-eosin, HE）染色后用于组织学评估。病理学评估由具有丰富肺癌诊断经验的病理学医师进行独立判读。

### 1.2 单细胞样本制备与转录组文库构建和测序

在无菌条件下，使用预冷的RPMI-1640培养基（含0.04% BSA）洗涤新鲜组织样本，并将其剪碎至约0.5 mm³大小。组织块在含0.2%胶原酶I的消化液中于37 ^o^C消化30-60 min，并不时轻柔混匀。消化后的细胞悬液经40 μm细胞筛过滤，离心后进行红细胞裂解，并用培养基洗涤。最终获得的单细胞悬液使用0.04% BSA的RPMI-1640重悬，并检测细胞浓度及存活率。将细胞浓度调整至700-1200个细胞/μL，按照MobiCube高通量单细胞3′转录组试剂盒（V2.1）说明，在MobiNova-100微流控平台上完成单细胞捕获与文库构建。文库采用Illumina NovaSeq 6000平台进行PE150高通量测序。

### 1.3 scRNA-seq数据处理与质量控制

scRNA-seq数据采用Cell Ranger软件（版本3.1.0，10× Genomics）进行初步处理，并比对至人类参考基因组GRCh38。使用Cell Ranger生成的过滤特征条形码表达矩阵作为后续分析的输入数据。为去除低质量细胞和潜在技术噪音，基于以下标准对细胞和基因进行质量控制：剔除检测到基因数<200或>50,000的细胞、独特分子标识符（unique molecular identifiers, UMI）总数<500或>50,000的细胞、UMI数量位于样本前1%的细胞（以减少潜在多细胞包裹的影响）以及线粒体基因表达比例>20%的细胞。同时，去除在<3个细胞中表达的基因。质量控制完成后，使用Seurat R包（版本5.3.0）对过滤后的表达矩阵进行下游分析，并通过NormalizeData函数对细胞表达数据进行归一化处理，以消除测序深度差异带来的影响。

### 1.4 细胞类型鉴定与特征分析

针对每个独立样本，采用Seurat分析流程对归一化后的表达矩阵进行降维和无监督聚类分析。首先，为满足下游分析需要，使用FindVariableGenes函数筛选2000个高变基因。随后，利用ScaleData函数对表达矩阵进行标准化处理。各个样本的表达矩阵按样本进行合并，并重新进行归一化和标准化。接着，基于高变基因使用Seurat R包中的RunPCA函数进行主成分分析（principal components analysis, PCA）以实现降维，参数设置为npcs=50。在主要分析中，通过基于图结构的聚类方法，使用FindNeighbors和FindClusters函数获得无监督的细胞聚类结果。为直观展示细胞聚类情况，采用RunUMAP函数进行一致流形近似与投影（uniform manifold approximation and projection, UMAP）可视化。主要细胞群体首先通过初步聚类获得，随后结合既往文献报道的经典细胞标志基因对其进行注释。表达多种主要细胞类型标志基因的细胞被视为潜在双细胞，并在各聚类中分别予以剔除。使用R软件包harmony（版本1.0）进行批次校正，以考虑样品特异性效应。各亚群的标志基因通过FindAllMarkers函数鉴定，采用Wilcoxon秩和检验进行统计分析，并将P_adj_<0.05的基因定义为该细胞亚群的特异性标志基因。

### 1.5 单细胞拷贝数变异（copy number variation, CNV）与克隆性分析

采用inferCNV算法对标注为上皮细胞的细胞群进行CNV分析，以识别具有大尺度染色体CNV的恶性细胞。在分析过程中，首先去除在参考细胞中平均读取数<0.1的基因。以T细胞和自然杀伤（natural killer, NK）细胞作为参考细胞群，利用inferCNV R包（版本1.10.1）对恶性上皮细胞进行鉴定。恶性细胞与非恶性细胞的定义标准与既往文献报道的方法一致^[13]^。其次，采用inferCNV R包中的亚克隆分析方法，解析每位患者的亚克隆CNV架构，并通过系统发育树形式进行可视化，以展示肿瘤的克隆性及演化过程。

### 1.6 以细胞间通讯CellChat软件包构建肿瘤内通信网络

以CellChatDB.human评估TME细胞亚群与癌细胞之间的信号通路输入和输出。接下来，使用computeCommunProPathway和aggregateNet函数计算小区间通信网络和通信强度。在不同细胞类型之间推断潜在的配体-受体相互作用，并分别构建不同样本的细胞通讯网络。通过比较不同患者信号通路的强度和组成差异，评估TME中细胞互作模式的变化。

### 1.7 功能富集分析

为系统解析不同细胞亚群的生物学特征，基于clusterProfiler软件包（v4.8.3）对不同疗效分组中特定细胞类型的差异基因进行京都基因与基因组百科全书（Kyoto Encyclopedia of Genes and Genomes, KEGG）和基因本体论（Gene Ontology, GO）富集分析，GO富集分析主要从生物学过程（biological process, BP）、细胞组分（cellular component, CC）、分子功能（molecular function, MF）3个维度进行分析。

### 1.8 统计学分析

所有统计分析和结果展示均使用R软件（版本4.4.1）完成。连续变量比较采用Wilcoxon秩和检验。单细胞差异表达分析采用Seurat内置的Wilcoxon秩和检验，多重比较校正采用Benjamini Hochberg方法，P_adj_<0.05认为差异具有统计学意义。GO和KEGG富集分析中，以P_adj_或错误发现率（false discovery rate, FDR）<0.05为显著富集。基因集变异分析（Gene Set Variation Analysis, GSVA）评分、功能模块评分及其他细胞状态相关评分的组间比较采用Wilcoxon秩和检验。统计显著性水平设定为P或P_adj_<0.05。

## 2 结果

### 2.1 新辅助治疗前后肺鳞癌患者的影像学变化和病理学特征分析变化及组织的scRNA-seq图谱概览

本研究首先评估了2例肺鳞癌患者新辅助免疫联合化疗后的病理学及影像学变化。HE染色显示，2例患者的术后肿瘤组织均呈现出明显的治疗相关改变：肿瘤细胞密度整体较低，残余肿瘤细胞散在分布，并伴组织结构破坏及炎性细胞浸润（图1A）。病例1为主要病理缓解（major pathological response, MPR），即残存活性肿瘤细胞<10%，病例2为部分病理缓解（partial pathological response, PPR），即残存活性肿瘤细胞10%-30%。影像学评估显示，2例患者在接受新辅助免疫联合化疗后肿瘤体积均出现不同程度变化（图1B）。病例1肿瘤明显缩小，符合PR标准。病例2仅轻度缩小，符合SD标准。2例患者的病理缓解程度与影像学评估结果并不完全一致。基于此，进一步对术后肿瘤组织进行scRNA-seq分析，以解析治疗后TME的变化特征。

**图1:**
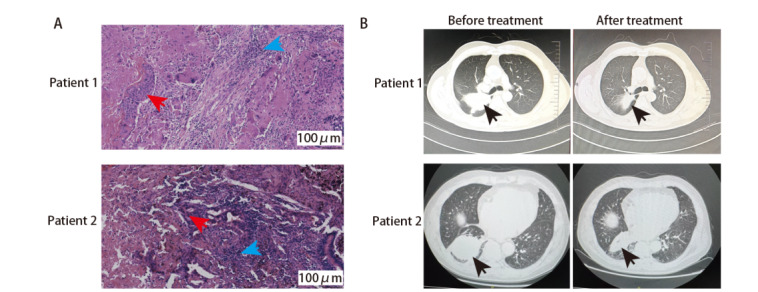
2例肺鳞癌患者新辅助治疗前后的CT图像及术后切除肿瘤组织的HE染色代表性图像。A：肿瘤细胞密度降低，残余肿瘤细胞散在分布，伴随炎性细胞浸润及组织重塑相关改变。红色箭头指的是肿瘤细胞；蓝色箭头指的是炎性淋巴细胞；B：病例1：治疗前肿瘤最大横截面为70 mm×65 mm，完成新辅助免疫联合化疗后缩小至46 mm×30 mm；病例2：治疗前肿瘤最大横截面为88 mm×64 mm，治疗后为80 mm×60 mm。箭头所指为肿瘤所在位置。所有测量均基于同一影像学模式下肿瘤最大横截面进行评估。

scRNA-seq分析共获得24,531个通过质控的细胞，无监督聚类识别出26个细胞簇（图2A），并根据经典标志基因区分出上皮细胞、髓系细胞、T细胞、成纤维细胞、内皮细胞、NK细胞、B细胞、浆细胞及肥大细胞等主要细胞类型（图2C）。2例患者肿瘤组织的UMAP分布如图2B所示。病例1与病例2在细胞分布及组成上存在明显差异（图2D-2E）。具体而言，病例1的细胞类型构成更为多元，上皮细胞、髓系细胞与T细胞均占一定比例；相比之下，病例2以髓系细胞为主要细胞群，上皮细胞占比降低，并伴随内皮细胞等基质相关细胞比例相对增加。本研究进而对各主要细胞群的代表性标志基因进行了展示。结果显示：不同细胞类型均表达其相应的经典标志分子，如上皮细胞标志基因、内皮细胞标志基因、成纤维细胞标志基因及免疫细胞相关分子等，从而验证了细胞类型注释的可靠性和准确性（图2F）。

**图2:**
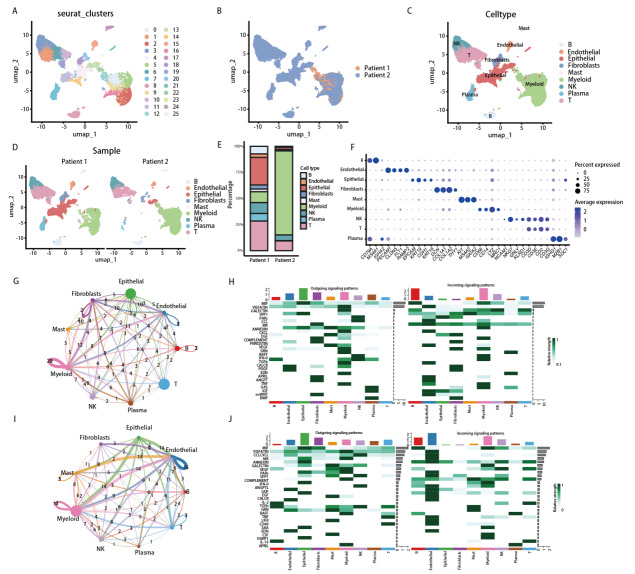
单细胞表征肺鳞癌患者新辅助免疫联合化疗后的肿瘤微环境图谱。A：所有细胞的UMAP降维可视化，共分为26簇；B：按样本来源的两组UMAP分布；C：主要细胞类型注释的UMAP图（包括上皮细胞、髓系细胞、T细胞、B细胞、NK细胞、成纤维细胞、内皮细胞、肥大细胞与浆细胞等）；D：按不同患者展示的UMAP；E：病例1与病例2样本中主要细胞类型比例图；F：用于主要细胞类型注释的代表性标志基因表达点图；G、H：病例1样本的细胞间通讯网络及信号输入、输出模式分析；I、J：病例2样本的细胞间通讯网络及信号输入、输出模式分析。

进一步应用Cellchat包分析了两组数据的细胞通讯，基于配体-受体推断分析分别构建了病例1和病例2样本的细胞间通讯网络。病例1整体通讯网络分析显示：上皮细胞、髓系细胞及成纤维细胞与多种免疫细胞之间存在广泛的信号交流，形成相对复杂的细胞互作网络，整体以CXC趋化因子（C-X-C motif chemokine ligand, CXCL）、表皮生长因子（epidermal growth factor, EGF）、ANNEXIN等炎症趋化相关通路为主（图2G-2H）。而病例2以内皮细胞和髓系细胞为主要信号枢纽，CXCL、分泌型磷蛋白1（secreted phosphoprotein 1, SPP1）、血管内皮生长因子（vascular endothelial growth factor, VEGF）、巨噬细胞移动抑制因子（macrophage migration inhibitory factor, MIF）及肿瘤坏死因子（tumor necrosis factor, TNF）等与趋化、炎症维持、血管生成和免疫调节相关的信号通路更为活跃（图2I-2J）。以上结果显示2例患者在接受新辅助免疫联合化疗后，其TME在细胞组成、空间分布及整体通讯格局上呈现出明显不同的重塑特征。

### 2.2 肿瘤组织内上皮细胞亚群的异质性及恶性特征的单细胞解析

为进一步解析新辅助免疫治疗后肺鳞癌肿瘤上皮细胞的异质性，对上皮细胞进行提取再聚类。二维t-分布随机邻居嵌入（t-distributed stochastic neighbor embedding, t-SNE）图识别出多个转录特征不同的亚群（图3A）。为区分恶性与非恶性上皮细胞，应用inferCNV对上皮细胞进行了分析（图3B）。以T细胞和NK细胞为基准，各亚群的总CNV评分中有4群评分较高组（图3C），其中高CNV亚群被推断为恶性上皮细胞，而低CNV亚群则更可能代表非恶性上皮细胞。结合转录特征及CNV评分，上皮细胞被划分为恶性细胞、Club细胞、纤毛细胞及肺泡II型上皮细胞（alveolar epithelial type II cell, AT2）等亚群（图3D）。不同亚型均证实表达各自特征性标志基因：如恶性上皮细胞表达EPCAM、KRT19等上皮相关基因；Club细胞高表达SCGB3A1、SCGB3A2等分泌相关基因；纤毛细胞特异性表达FOXJ1、TPPP3、DNAH9；AT2细胞则富集表达SFTPC、SFTPB、SFTPA1等肺泡相关基因（图3E），进一步证实了该亚群注释的可靠性。比较良/恶性上皮细胞占比发现，病例1肿瘤组织中恶性上皮细胞占比较高，为51%，而病例2则以非恶性上皮细胞占比为主（图3F）。2例样本中恶性与非恶性上皮细胞比例存在差异。鉴于inferCNV分析主要用于辅助识别恶性特征，相关比例结果需结合病理及取材背景综合解读。进而基于配体-受体分析上皮细胞在TME中的信号输出特征显示：在不同患者中，其网络结构和相互作用的强度存在明显差异（图3G）。这些结果提示新辅助治疗后肿瘤上皮细胞在恶性特征及细胞通讯模式上存在明显个体差异，可能与不同患者的疗效反应有关。

**图3:**
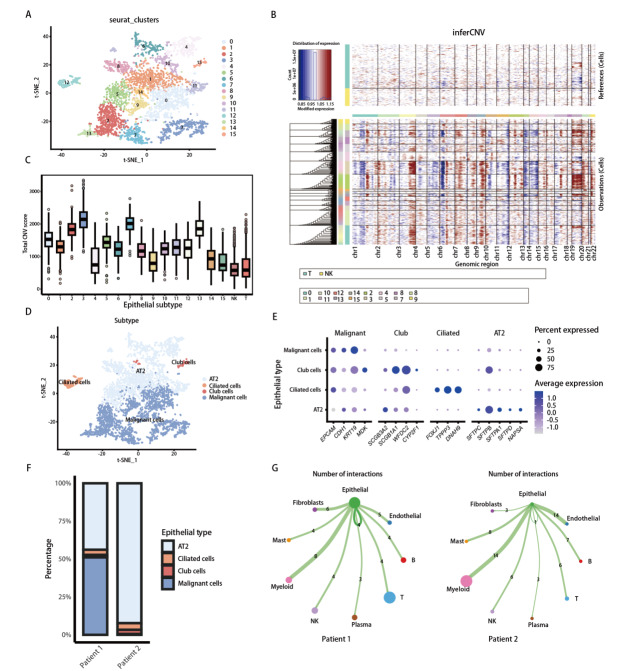
上皮细胞异质性及恶性特征的单细胞解析。A：t-SNE图展示了16簇提取的上皮细胞；B：inferCNV包展示热图，显示不同上皮细胞簇的CNV模式；C：各上皮细胞簇的总CNV评分；D：上皮细胞亚型注释（恶性上皮细胞、AT2细胞、Club细胞、纤毛细胞等）的可视化；E：上皮细胞亚型及恶性相关标志基因表达点图；F：2例样本中上皮细胞亚型的组成比例；G：以上皮细胞为中心的细胞通讯网络。

### 2.3 髓系细胞亚群的组成差异及免疫调节相关特征分析

为了深入研究髓系细胞的差异，对髓系细胞进行提取和再聚类鉴定出巨噬细胞、单核细胞、中性粒细胞及树突状细胞等亚群（图4A）。如图4B髓系细胞比例统计所示，2例患者在髓系亚群的组成及相对丰度上亦存在明显差异，病例1样本中单核细胞及树突状细胞比例相对更高，而病例2样本中巨噬细胞和中性粒细胞占比较高（图4C）。同样，各亚群均表达相应经典标志基因，支持细胞注释的可靠性（图4D）。

**图4:**
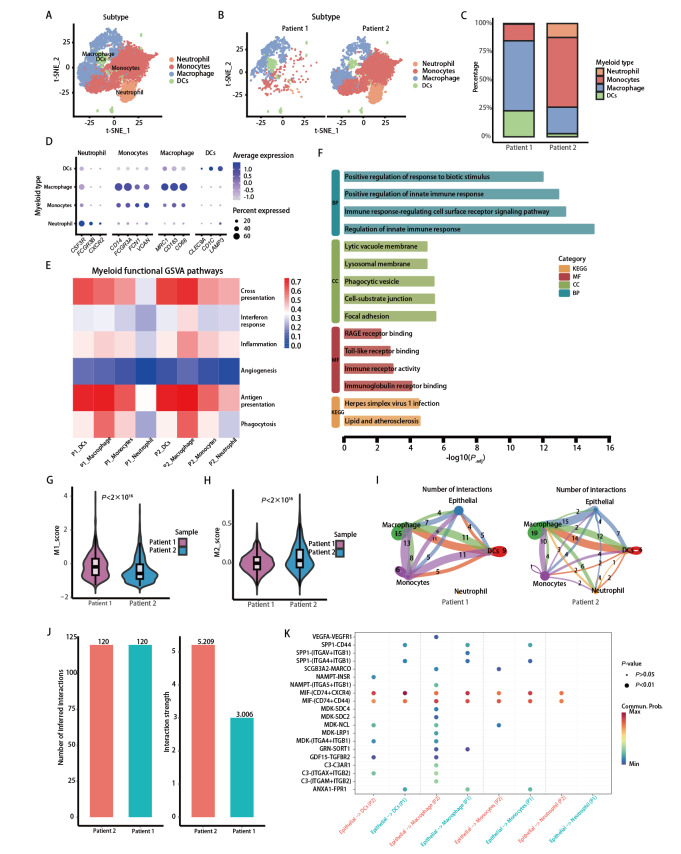
髓系细胞亚群、功能状态及免疫调节相关互作特征。A：髓系细胞提取后再聚类的t-SNE图，可区分巨噬细胞、单核细胞、中性粒细胞与树突状细胞等亚群；B：病例1与病例2展示的髓系细胞分布；C：不同样本中髓系细胞亚群的组成比例；D：髓系细胞亚群代表性标志基因表达点图；E：GSVA功能通路评分热图；F：髓系细胞差异基因的GO与KEGG富集分析；G、H：不同样本中髓系细胞M1与M2相关评分的小提琴图；I：不同样本中髓系细胞相关的细胞通讯网络；J、K：上皮细胞与髓系细胞互作的通路、配体-受体分析与量化展示，突出免疫调节相关信号轴。

随后对髓系细胞进行基因表达及通路富集分析。GSVA结果显示，不同髓系亚群在多条功能通路上呈现差异性富集特征，包括炎症反应、抗原呈递、吞噬作用及血管生成等过程（图4E）。病例2样本中的髓系细胞在抗原处理、先天免疫调控及炎症相关通路上呈现持续激活状态，且同时伴随干扰素反应减弱及血管生成相关信号增强。图4F所示GO与KEGG富集结果显示，病例2样本中髓系细胞富集到慢性炎症调控、免疫调节及代谢重编程相关通路。而病例1样本中的髓系细胞则表现出更均衡的免疫激活特征。这些结果初步揭示新辅助免疫治疗中髓系细胞在免疫调控和组织重塑过程中发挥着重要的作用。

此外，为了进一步探讨巨噬细胞在免疫新辅助治疗中的变化，对2个样本中的巨噬细胞进行M1和M2打分。结果显示，病例1样本中髓系细胞的M1相关评分明显高于病例2样本（图4G），而病例2样本则表现为M2极化趋势（图4H）。该结果提示病例1样本中的巨噬细胞更偏向炎症激活和效应相关特征，而病例2样本中的巨噬细胞则可能更倾向于呈现一种促肿瘤和免疫调节相关的功能状态。在细胞间相互作用层面，配体-受体分析显示2例样本中髓系细胞相关的通讯网络在整体结构及信号指向方面存在差异（图4I）。进一步比较不同患者样本中髓系细胞中介导的信号通路数量及整体通讯强度，发现其在多条与免疫调节、炎症反应及细胞迁移相关的信号轴上的参与程度并不一致（图4J）。

配体-受体分析亦进一步揭示了2例样本在上皮细胞-髓系细胞通讯层面的差异。多条免疫抑制及促进TME构建相关的信号轴在病例2样本中呈现相对更高的活跃程度（图4K）。其中，上皮细胞与巨噬细胞之间的SPP1-CD44及SPP1-整合素（ITGAV/ITGA4-ITGB1）信号轴在病例2样本中显著增强，提示TME中SPP1^+^肿瘤肿瘤相关巨噬细胞（tumor associated macrophage, TAM）相关的黏附与迁移信号可能更加活跃。而MIF-CD74-CXCR4/CD44轴在病例2样本中持续活跃提示上皮细胞可能通过MIF相关信号参与免疫抑制性微环境的维持。这些信号轴的相对增强与病例2样本中髓系细胞M2相关评分升高的结果相一致。总之，从细胞组成、功能状态及细胞间通讯多个层面展开分析提示新辅助免疫治疗后肺鳞癌髓系细胞呈现显著异质性，表现在不同个体中髓系细胞所介导的免疫调节网络存在明显差异。

### 2.4 T淋巴细胞亚群异质性及功能状态差异分析

为解析新辅助免疫治疗后肺鳞癌TME中适应性免疫细胞的状态差异，对肿瘤浸润T细胞进行了再聚类分析发现存在多个具有明确功能指向的T淋巴细胞亚群。除CD4^+^ Naive、CD4^+^ Th与CD4^+^ Treg外，CD8^+^ T细胞进一步分化为效应（CD8^+^ eff）、效应记忆（CD8^+^ EM）以及耗竭相关群体（CD8^+^ Ex），并可识别出一定比例的γδ T细胞（图5A）。在不同样本中的T细胞组成呈现出不同的亚群构成（图5B）。病例1样本中CD4^+^ Th及CD8^+ ^eff亚群所占比例相对更高；而病例2样本中免疫调节相关的CD4^+^ Treg以及终末耗竭相关的CD8^+^ Ex亚群占比明显增加，效应性CD8^+^ T细胞比例相对降低。各亚群的标志分子表达进一步支持上述注释与功能定位（图5C）。与此同时，差异基因富集结果集中在细胞趋化、免疫反应调控与趋化因子信号通路等通路上（图5D），提示T细胞状态差异不仅体现在效应分子表达，也与TME中的募集与迁移过程密切关联。

**图5:**
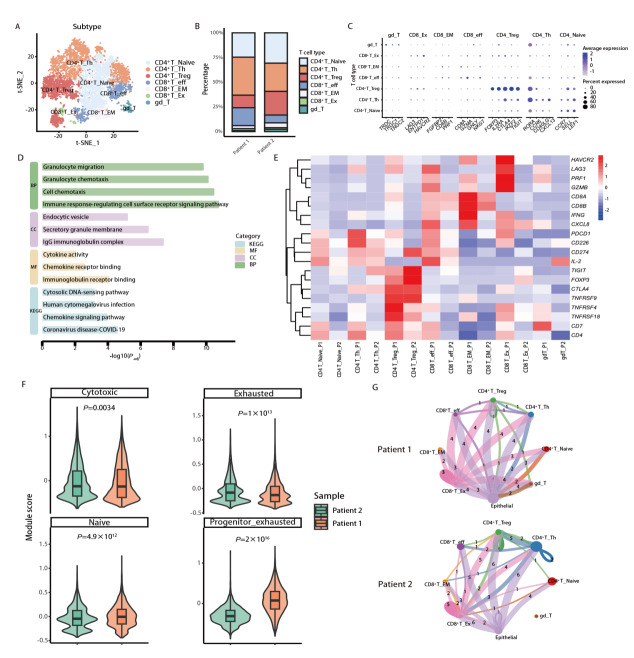
T 细胞异质性、功能状态差异及互作网络重塑差异分析。A：T细胞提取后再聚类的t-SNE图，识别出CD4^+^初始、CD4^+^ Th、CD4^+^ Treg、CD8^+^效应、CD8^+^效应记忆、CD8^+^耗竭及γδT等亚群；B：2例样本中各T细胞亚群的组成比例；C：T细胞功能状态相关标志基因表达点图；D：T细胞差异基因的功能富集分析；E：不同样本的关键免疫检查点相关基因表达热图；F：不同样本中细胞毒性、耗竭、初始及前体耗竭等模块评分的小提琴图；G：不同样本中上皮细胞与T细胞相关的细胞通讯网络。

比较不同患者，各T细胞亚群的功能相关基因呈现出更为清晰的“组内偏移”模式（图5E）。在CD8^+^ eff与CD8^+^ EM亚群中，病例1样本整体更具效应输出：细胞毒性相关分子（如PRF1、GZMB）及效应相关信号（如IFNG）的表达更为突出；相比之下，病例2样本在上述效应分子上相对减弱，同时与功能受限相关的分子呈现更明显的上调趋势，表明2例患者的CD8^+^ T细胞在效应分子表达及耗竭相关转录特征的分布方式上呈现出不同的状态组合。而在CD8^+^ Ex亚群中，病例1样本组的前体耗竭相关分子同样表达更为集中，尤其是HAVCR2、LAG3以及PDCD1等检查点分子显示出更强的上调倾向。提示其T细胞群体在不同亚群层面同时呈现效应功能维持与耗竭相关转录特征的并存状态。进一步对功能模块评分进行定量评估发现，与病例1样本相比，病例2样本的耗竭模块评分略高；而病例1样本的前体耗竭模块评分显著上升（图5F）。提示2例患者在T细胞耗竭相关分化状态上存在差异。与此同时，两者在整体细胞毒性程序上的评分水平相当。细胞互作分析进一步补充了2例样本间的免疫生态差别，病例1样本中T细胞与上皮细胞及其他免疫细胞之间呈现相对更为活跃的互作结构（图5G）。综合上述结果，T细胞亚群构成、关键功能分子表达与模块评分的差异共同描绘出新辅助免疫治疗后，不同样本在适应性免疫细胞的分化阶段分布及耗竭相关程序偏移上呈现出不同的状态谱。

## 3 讨论

近年来，新辅助免疫联合化疗逐渐成为可切除NSCLC的重要治疗策略，其优势不仅体现在潜在的肿瘤降期和切除率提升，更在于其能够在肿瘤仍原位存在、抗原负荷和TME结构尚未被手术的条件下，诱导并放大全身及局部抗肿瘤免疫反应^[14]^。然而，患者对治疗的反应存在显著异质性，其潜在的细胞与TME基础尚不清楚，疗效评估多依赖于影像学检查，且面临精准的生物标志物缺乏等问题^[15]^。本研究纳入2例接受新辅助免疫治疗后手术切除的肺鳞癌患者，尽管影像学检查对患者疗效的评估为PR和SD，但术后病理均提示残余活性肿瘤细胞比例较低，分别达到MPR和PPR。进一步通过单细胞转录组分析发现，2例患者在治疗后TME的细胞组成、功能状态及细胞间通讯层面均存在明显异质性，其中病例1呈现免疫细胞富集和炎症激活特征，病例2则表现为以髓系及内皮信号重塑为主、伴随更明显免疫调节和血管生成相关特征。这提示，新辅助免疫联合化疗后的疗效差异，可能不仅体现为肿瘤负荷变化，更与治疗后TME重塑方向密切关联。

TAM是TME的重要组成部分^[16]^。TME中，髓系细胞作为连接先天免疫与适应性免疫的重要组成部分，参与抗原呈递、炎症反应调控及免疫应答塑形等多种关键生物学过程^[17]^。本研究中，髓系细胞的差异是2例患者治疗后TME重塑最突出的特征之一。病例2样本中巨噬细胞和中性粒细胞比例较高，且GSVA、GO和KEGG分析均提示其髓系细胞更显著富集于炎症维持、免疫调节及血管生成相关通路。同时，M1/M2特征评分显示，病例2更倾向于M2相关表型，而病例1则保留更高的M1相关评分。更关键的是，细胞通讯分析在病例2样本中捕捉到以SPP1为核心的上皮细胞-巨噬细胞互作增强（如SPP1-CD44及SPP1-整合素复合体相关轴），这一信号组合常见于TAM中具有组织重塑、黏附迁移及免疫抑制特征的亚群，并被多组学研究中被反复报道与免疫排斥表型、血管生成及侵袭相关的TME特征相一致^[18]^。与此同时，病例2样本中MIF-CD74-CXCR4/CD44等互作轴持续活跃，结合补体相关互作（如C3-C3AR1）以及MDK相关旁分泌信号，整体描绘出一种更偏向维持髓系细胞募集、存活与免疫调节功能的网络结构^[19]^。综合上述结果，病例2样本中的髓系细胞更倾向于将持续的先天免疫刺激转化为促肿瘤和免疫抑制相关信号输出，其中以SPP1^+^/M2样TAM相关通讯最具代表性。这一特征可能构成其免疫抑制性TME的重要基础，并在一定程度上与其影像学仅表现为SD相对应。

淋巴细胞在抗肿瘤免疫中发挥着关键作用^[20]^。除髓系细胞外，T细胞状态差异同样构成2例患者治疗后TME异质性的另一关键层面。本研究显示，病例1中CD4^+^ Th、CD8^+^ eff及CD8^+^ EM细胞比例较高，且PRF1、GZMB、IFNG等效应相关分子表达更为突出；病例2则表现为CD4^+^ Treg和CD8^+^ Ex比例增加，提示其适应性免疫反应更倾向于免疫调节和功能受限状态。值得注意的是，功能模块评分显示，病例2终末耗竭相关程序略占优势，而病例1则富集更多前体耗竭相关模块。这些结果提示，新辅助免疫治疗后，肿瘤浸润T细胞并非简单地表现为效应增强或功能衰竭，而可能沿不同分化阶段发生重塑。近年来的研究^[21]^逐渐表明，T细胞耗竭并非单一、终末的功能衰竭状态，而是一个具有分化层级和动态转化特征的连续谱系。在这一框架下，前体耗竭T细胞被认为代表了一类介于初始和效应状态与终末耗竭状态之间的中间群体，其通常保留一定的增殖潜能、转录可塑性及对免疫调控信号的响应能力，而终末耗竭T细胞则更多表现为持续抗原刺激下形成的功能受限状态^[22]^。因此，本研究中病例1虽存在一定耗竭相关特征，但仍保留较强效应功能和更活跃的T细胞互作网络；而病例2则更突出地表现为终末耗竭及免疫抑制程序增强。然而，即便在探索性框架下，SPP1^+^巨噬细胞相关互作增强与CD8^+^ T细胞耗竭程序上调在病例2样本中的共现，共同构成治疗后免疫抑制性TME重塑的两个相互关联的层面。

此外，本研究中影像学评估、病理缓解与单细胞层面的结果并不完全一致，提示不同评估手段反映的是治疗反应的不同维度：影像学主要体现病灶体积变化，病理评估侧重残余活性肿瘤比例，而单细胞测序则揭示治疗后TME的组成与功能状态。因此，单纯依赖影像学可能难以全面反映真实疗效。当然，本研究仍存在明显局限。首先，病例数仅2例，样本量较小，研究结论仍属于探索性观察，尚不足以进行稳健的群体推断。其次，本研究为术后单时间点的横断面观察，只能呈现治疗后某一时点的TME状态，无法动态追踪治疗前后及围手术期不同阶段细胞组成与功能状态的连续演变，也难以直接建立细胞互作变化与疗效差异之间的因果关系。此外，部分细胞比例及恶性特征识别结果还可能受到取材区域、组织成分及单细胞捕获效率等因素的影响。因此，后续研究将进一步扩大病例数，并增加观察时间点，在治疗前、治疗中及手术后多个阶段连续采样；同时结合空间转录组学、免疫组织化学及功能实验，对SPP1^+^ TAM的空间定位、细胞邻域关系及其与T细胞状态转化之间的联系进行验证，以期更系统地揭示新辅助免疫联合化疗后肺鳞癌TME重塑规律，并为疗效评估和潜在生物标志物筛选提供更详实的依据。

## References

[1] Sung H, Ferlay J, Siegel RL, et al. Global cancer statistics 2020: GLOBOCAN estimates of incidence and mortality worldwide for 36 cancers in 185 countries. CA Cancer J Clin, 2021, 71(3): 209-249. doi: 10.3322/caac.21660 33538338

[2] Herbst RS, Morgensztern D, Boshoff C. The biology and management of non-small cell lung cancer. Nature, 2018, 553(7689): 446-454. doi: 10.1038/nature25183 29364287

[3] Travis WD, Brambilla E, Nicholson AG, et al. The 2015 World Health Organization classification of lung tumors: Impact of genetic, clinical and radiologic advances since the 2004 classification. J Thorac Oncol, 2015, 10(9): 1243-1260. doi: 10.1097/jto.0000000000000630 26291008

[4] Reck M, Rodríguez-Abreu D, Robinson AG, et al. Pembrolizumab versus chemotherapy for PD-L1-positive non-small-cell lung cancer. N Engl J Med, 2016, 375(19): 1823-1833. doi: 10.1056/NEJMoa1606774 27718847

[5] Forde PM, Spicer J, Lu S, et al. Neoadjuvant Nivolumab plus chemotherapy in resectable lung cancer. N Engl J Med, 2022, 386(21): 1973-1985. doi: 10.1056/NEJMoa2202170 35403841 PMC9844511

[6] Riely GJ, Wood DE, Ettinger DS, et al. Non-small cell lung cancer, version 4.2024, NCCN clinical practice guidelines in oncology. J Natl Compr Canc Netw, 2024, 22(4): 249-274. doi: 10.6004/jnccn.2204.0023 38754467

[7] Parisi C, Abdayem P, Tagliamento M, et al. Neoadjuvant immunotherapy strategies for resectable non-small cell lung cancer (NSCLC): Current evidence among special populations and future perspectives. Cancer Treat Rev, 2024, 131: 102845. doi: 10.1016/j.ctrv.2024.102845 39442290

[8] Hellmann MD, Chaft JE, William WN Jr, et al. Pathological response after neoadjuvant chemotherapy in resectable non-small-cell lung cancers: Proposal for the use of major pathological response as a surrogate endpoint. Lancet Oncol, 2014, 15(1): e42-e50. doi: 10.1016/s1470-2045(13)70334-6 24384493 PMC4734624

[9] Binnewies M, Roberts EW, Kersten K, et al. Understanding the tumor immune microenvironment (TIME) for effective therapy. Nat Med, 2018, 24(5): 541-550. doi: 10.1038/s41591-018-0014-x 29686425 PMC5998822

[10] Zheng J, Yan Z, Wang R, et al. NeoPred: dual-phase CT AI forecasts pathologic response to neoadjuvant chemo-immunotherapy in NSCLC. J Immunother Cancer, 2025, 13(5): e011773. doi: 10.1136/jitc-2025-011773 40449955 PMC12163334

[11] Papalexi E, Satija R. Single-cell RNA sequencing to explore immune cell heterogeneity. Nat Rev Immunol, 2018, 18(1): 35-45. doi: 10.1038/nri.2017.76 28787399

[12] Cui X, Liu S, Song H, et al. Single-cell and spatial transcriptomic analyses revealing tumor microenvironment remodeling after neoadjuvant chemoimmunotherapy in non-small cell lung cancer. Mol Cancer, 2025, 24(1): 111. doi: 10.1186/s12943-025-02287-w 40205583 PMC11980172

[13] Wang Z, Li Z, Zhou K, et al. Deciphering cell lineage specification of human lung adenocarcinoma with single-cell RNA sequencing. Nat Commun, 2021, 12(1): 6500. doi: 10.1038/s41467-021-26770-2 34764257 PMC8586023

[14] Uprety D, Mandrekar SJ, Wigle D, et al. Neoadjuvant immunotherapy for NSCLC: Current concepts and future approaches. J Thorac Oncol, 2020, 15(8): 1281-1297. doi: 10.1016/j.jtho.2020.05.020 32522713

[15] Kang J, Zhang C, Zhong WZ. Neoadjuvant immunotherapy for non-small cell lung cancer: State of the art. Cancer Commun (Lond), 2021, 41(4): 287-302. doi: 10.1002/cac2.12153. 33689225 PMC8045926

[16] Mantovani A, Marchesi F, Malesci A, et al. Tumour-associated macrophages as treatment targets in oncology. Nat Rev Clin Oncol, 2017, 14(7): 399-416. doi: 10.1038/nrclinonc.2016.217 28117416 PMC5480600

[17] Ngambenjawong C, Gustafson HH, Pun SH. Progress in tumor-associated macrophage (TAM)-targeted therapeutics. Adv Drug Deliv Rev, 2017, 114: 206-221. doi: 10.1016/j.addr.2017.04.010 28449873 PMC5581987

[18] Zhang Q, He Y, Luo N, et al. Landscape and dynamics of single immune cells in hepatocellular carcinoma. Cell, 2019, 179(4): 829-845.e20. doi: 10.1016/j.cell.2019.10.003 31675496

[19] Camacho Meza G, Avalos Navarro G, De La Cruz Mosso U, et al. Macrophage migration inhibitory factor: Exploring physiological roles and comparing health benefits against oncogenic and autoimmune risks (Review). Int J Mol Med, 2025, 56(4): 149. doi: 10.3892/ijmm.2025.5590 40682854 PMC12306601

[20] Fridman WH, Pagès F, Sautès-fridman C, et al. The immune contexture in human tumours: Impact on clinical outcome. Nat Rew Cancer, 2012, 12(4): 298-306. doi: 10.1038/nrc3245 22419253

[21] Wherry EJ, Kurachi M. Molecular and cellular insights into T cell exhaustion. Nat Rew Immunol, 2015, 15(8): 486-499. doi: 10.1038/nri3862 PMC4889009 26205583

[22] Liu Z, Zhang Y, Ma N, et al. Progenitor-like exhausted SPRY1(+)CD8(+) T cells potentiate responsiveness to neoadjuvant PD-1 blockade in esophageal squamous cell carcinoma. Cancer Cell, 2023, 41(11): 1852-1870.e9. doi: 10.1016/j.ccell.2023.09.011 37832554

